# LEGAL BASES FOR DISCLOSING CONFIDENTIAL PATIENT INFORMATION FOR PUBLIC HEALTH: DISTINGUISHING BETWEEN HEALTH PROTECTION AND HEALTH IMPROVEMENT

**DOI:** 10.1093/medlaw/fwv018

**Published:** 2015-05-20

**Authors:** Mark J. Taylor

**Affiliations:** School of Law, The University of Sheffield, Sheffield, UK

**Keywords:** Public health improvement, Patient confidential information, SI 2002/1438

## Abstract

The disclosure of confidential patient data without an individual's explicit consent should be for purposes that persons have reason to both expect and accept. We do not currently have the required level of clarity or consistency in understanding regarding the disclosure of confidential patient information for public health purposes to support effective public dialogue. The Health Service (Control of Patient Information) Regulations 2002 establish a legal basis in England and Wales for data to be disclosed for public health purposes without patient consent. Under the Regulations, there is more than one potential route towards lawful processing: Data may be processed for public health purposes under both Regulations 3 and 5. The alternatives have different safeguards and conditions attached, and their respective applicability to processing for purposes of public health improvement is currently unclear and subject to review. Beyond the need for clarity regarding the safeguards applicable to processing for particular public health purposes, there are reasons to prefer recognition that Regulation 5 is the most appropriate legal basis for disclosure when the purpose is public health improvement rather than public health protection. Where health improvement, rather than protection, is the aim, there is no justification for discarding the additional safeguards associated with processing under Regulation 5.

## I. INTRODUCTION

The analysis of routinely gathered health-care data at a population level, linked with other digital data, can provide tremendous opportunities to better understand, and improve, public health. As more data are routinely gathered by health-care providers, and the technical possibilities regarding the processing, linkage, and analysis of that data improve and cheapen, there is growing potential for public benefit beyond improvement to an individual's direct care. This potential will only be realised if the necessary data can flow to persons who do not need it to deliver individual care and treatment. However, any use of individual health-care records for purposes beyond direct patient care, including for public health purposes, must be done with great care.^1^ There is already a ‘data deficit’ in the trust that individuals have in institutions, which consistently dips when individuals are asked about confidence that personal data will be used appropriately.^2^ In these circumstances, confidential health data should only flow for purposes beyond direct care that the public have both reason to expect and also reason to accept as appropriate. Assurance on appropriate use must be provided through proportionate and transparent safeguards and controls.^3^ However, in England and Wales, the Caldicott2 report last year identified ‘a lack of coherence in regulations across the public health arena’^4^ and called for greater clarity regarding the legal bases for the disclosure of confidential patient information for particular public health purposes, including specifically those outside health protection and cancer registration.

If public trust in the confidentiality of the health record is lost, and any confidence that sensitive health information is only to be used appropriately is undermined, then there is a concern that patients will not be candid with health-care professionals.^5^ Maintaining confidence that data will only be used in appropriate ways—ensuring ‘no surprises’^6^—requires an ongoing dialogue with members of the public about the terms upon which confidential data are used. Such a dialogue urgently requires clarity regarding the relevant legal bases of disclosure. Without a uniform understanding across different professionals regarding the relevant legal basis of any data flow, it is impossible to be consistently clear about the applicable safeguards and controls. A lack of clarity and consistent transparency compromises opportunities to let people know what to expect and undermines any ability to provide assurance that data will *only* be used for purposes that persons have reason to accept.

In England and Wales, not only the need for ‘a more transparent dialogue’^7^ has been recognised, but the need for increased clarity and consistency of message is also currently being felt here particularly keenly.^8^ This article responds to the call for improved clarity regarding the legal bases for disclosure of confidential patient data for public health purposes. It articulates reasons for reliance upon specific parts of the Health Service (Control of Patient Information) Regulations 2002 to justify disclosure of confidential patient information for different public health purposes. In particular, it suggests that a distinction should be drawn between different parts of the regulations, namely Regulations 3 and 5, according to whether processing is for the purposes of public health *protection* or public health *improvement*. More clearly, aligning disclosure of confidential patient data for particular public health purposes with particular parts of the 2002 Regulations will promote greater uniformity in the application of controls and safeguards associated with the disclosure for these different kinds of public health purposes.

Further, the argument advanced is that a relatively narrow interpretation of Regulation 3, which would restrict it to purposes of public health *protection*, continues to be more consistent with the original intention behind the 2002 Regulations and broader principles of statutory interpretation. What is more, this interpretation—which would see Regulation 5 as the more appropriate basis for disclosure for the purposes of public health *improvement—*will better meet requirements under human rights legislation. A separate but related point is that it also importantly opens the space to realise the deliberative potential of discussion by the independent group that advises on the application of Regulation 5. None of this is to suggest that it may not be appropriate to disclose for the purposes of public health improvement under the 2002 Regulations. Rather, it implies that (1) there is a need to be clear whether, and if so when, disclosure for the purposes of public health improvement may be under Regulation 3 or 5, and (2) there are reasons to prefer disclosure for the purposes of public health improvement under Regulation 5. This discussion takes place in the shadow of a bigger discussion about the future of EU Data Protection. Without wanting to confuse a discussion of data protection law with the purpose and effect of the 2002 Regulations (which is essentially concerned with setting aside the Common Law Duty of Confidence), it is worth noting that some versions of proposed text for a future EU Data Protection Regulation may prevent some of the disclosures being described here.^9^ Clarifying the safeguards around such disclosures, demonstrating how they can take place with appropriate protection of individual privacy, may offer support to arguments that preventing such disclosures is not necessary to achieve appropriately robust protection.

## II. DISCLOSING CONFIDENTIAL PATIENT INFORMATION FOR PUBLIC HEALTH PURPOSES

In England and Wales, the use of confidential patient data is subject to a complex matrix of legal requirements. In addition to the requirements of the Data Protection Act 1998, which are not the focus of this article,^10^ there are duties owed under the Common Law Duty of Confidence. Under English Law, it is clear that despite any Duty of Confidence that may be owed in relation to patient data, there may be a legal basis for processing confidential patient information for public health purposes. The Health Protection (Notification) Regulations 2010 set aside the common law duties by a statutory duty imposed on registered medical practitioners to notify certain specified infectious diseases and contaminations to the proper officer of the local authority via Public Health England (PHE). In addition, diagnostic laboratories are required to provide positive diagnostic test results on specified diseases to PHE. Furthermore, the common law duties may be set aside under the Health Service (Control of Patient Information) Regulations 2002 (hereafter ‘the 2002 Regulations’). What needs to be clearer is *which* parts of the 2002 Regulations can be relied upon under which circumstances.

Regulation 3 of the 2002 Regulations can set aside the restrictions specifically associated with the Common Law Duty of Confidence when processing is intended to diagnose, control, prevent, or recognise trends in ‘communicable diseases and *other risks to public health*’ (emphasis added). Other parts of the 2002 Regulations, including a more generally applicable Regulation 5, can set aside the common law restrictions in other circumstances. Regulations 3 and 5 have different controls and safeguards associated with them. Consistent understanding of the scope of the phrase ‘other risks to public health’ is necessary for consistent description of the controls and safeguards that will apply to disclosure for different public health purposes. A narrow reading of ‘other risks to public health’ to date—restricting it to health protection—has undoubtedly placed restrictions on the flow of data for public health purposes. Prompted by the most recent Caldicott2 report, the current position is being reviewed by PHE.^11^

One of the reasons that a narrow reading of Regulation 3 places restrictions on the flow of data for public health purposes is that disclosure under Regulation 5 is associated with independent review by an advisory group. A standard requirement imposed by the group is that individual objection to disclosure should be respected in all but the most exceptional circumstances.^12^ Both independent review and the possibility for individual opt-out are safeguards that, in the context of research use at least, have been described as relevant to the acceptability of the disclosure of confidential patient data without patient consent.^13^ They may also constitute ‘warrants of trust’ relevant to ensuring that activity does not extend beyond that which has ‘social license’ irrespective of strict legal authority.^14^ That is to say, the safeguards associated with Regulation 5 may be relevant to the maintenance of public confidence in the disclosure of confidential patient information, without patient consent, for purposes of improving public health. There may be sound regulatory reasons for preferring reliance upon Regulation 5, particularly when addressing those applications of the 2002 Regulation likely to be the most controversial. While these are important points and I will return to them at the end, they are at best supplemental to the legal argument for preferring a narrow interpretation of Regulation 3. Before unpacking the argument in terms of legal rationality, however, it is necessary to say a little more about the legal context in which the 2002 Regulations operate and to explain the need for clarity regardless of which interpretation of Regulation 3 is eventually to be preferred.

### A. The Common Law Duty of Confidence and Public Health

The records of patients in England and Wales are not held centrally by the National Health Service (NHS). Instead, the various parts of the NHS hold their own records of patient care and treatment. Records are held separately by the General Practitioner (GP),^15^ NHS Hospital, NHS dentist, and many other providers of specialist, publicly funded or privately paid for patient care and treatment. Each of the health-care professionals operating within these different organisations owes a duty of confidentiality to his or her patients.^16^ This duty to protect *identifiable* patient data is rooted in professional ethics as well as common law.^17^ This duty protects patients from the inappropriate sharing of the confidential patient data and is generally to be regarded as a good thing. However, there have been concerns expressed in England that the controls applied to protect patient confidence can also inhibit the appropriate sharing of patient data. This concern has been expressed in relation to sharing for both direct care and secondary use (also known as indirect care) purposes.^18^

Stated in general terms, the Common Law Duty of Confidence recognises that those in receipt of confidential patient information are under a duty to hold and disclose that information in ways that respect a patient's ‘reasonable expectations of privacy’.^19^ This is typically interpreted to mean that

information that can identify individual patients, must not be used or disclosed for purposes other than healthcare without the individual's explicit consent, some other legal basis, or where there is a robust public interest or legal justification to do so.^20^

While this may be a defensible position in both law and principle, it is not always clear exactly what it requires in practice: There have been few cases considering the outer limits to the uses of information supportable on the basis of a consent to receive healthcare. For example, does an explicit consent to healthcare imply also that one consents to the use of identifiable information to audit the care or treatment received?^21^ Generally, the lack of precedent describing the distinction between reasonable and unreasonable expectations in this context can leave professionals understandably unclear on the boundaries between permitted sharing and unlawful disclosure. Reluctant to risk breaching any duty, the uncertainty can encourage a cautious approach.^22^ Just as the limits of implied consent are unclear, so the range of disclosures permissible due to a ‘robust public interest’ is not clearly defined.

The Common Law Duty of Confidence is rooted in the idea of ‘the public interest’. There is recognised to be a public interest (and not merely a private interest) in the preservation of an individual's right to confidence.^23^ However, at times, the public interest may require the disclosure of confidential information.^24^ The courts have been willing to support the unauthorised disclosure of confidential information to protect the public from a risk of harm. When balancing competing interests in the particular circumstances that gave rise to the case of *W* v *Edgell*,^25^ Lord Bingham found there to be one consideration that

weighs the balance of public interest decisively in favour of disclosure. It may be shortly put… A consultant psychiatrist who becomes aware, even in the course of a confidential relationship, of information which leads him, in the exercise of what the court considers a sound professional judgment, to fear that such decisions may be made on the basis of inadequate information and with a real risk of consequent danger to the public is entitled to take such steps as are reasonable in all the circumstances to communicate the grounds of his concern to the responsible authorities.^26^

Having identified a ‘real risk of consequent danger to the public’, the court found the public interest lay in Dr Edgell taking ‘reasonable steps’ to disclose confidential patient information contrary to his patient's expressed wish.^27^ The risk present need not to be associated with criminal activity or some other kind of misconduct.^28^ In *Malone* v *Commissioner of Police of the Metropolis* (*No. 2*), Sir Robert Megarry offered a relevant example:

There may be cases where there is no misconduct or misdeed but yet there is a just cause or excuse for breaking confidence. The confidential information may relate to some apprehension of an impending chemical or other disaster, arising without misconduct, of which the authorities are not aware, but which ought in the public interest to be disclosed to them.^29^

One can imagine a scenario in which, faced with an impending chemical disaster and a real risk of consequent danger to the public, any court required ‘to carry out a balancing operation between the public interest in maintaining confidence and the public interest in disclosure’^30^ would consider the balance to be firmly tipped towards disclosure. However, that scenario may be considered to be towards one end of a particular spectrum. It is difficult to anticipate precisely where the courts will find the tipping point in the face of different kinds of risk to public health. Risks themselves may vary according to magnitude, immediacy, and agency. Effective mitigation of risk may also varyingly depend upon different levels of disclosure, of different types of information of different levels of sensitivity, to different numbers of persons in different roles for different reasons. While we might anticipate that the courts support for limited disclosure to *protect* the public from ‘impending disaster’, would they support routine and widespread disclosure of sensitive data to undertake surveillance or planning for more common risks? The 2002 Regulations were, at least in part, a response to this lack of clarity regarding when public health-related activity could meet the common law thresholds for public interest disclosure.

In response to a concern that certain specific activities may not be carried out in ways that meet responsibilities under the Common Law Duty of Confidence, Parliament introduced a power to allow the Secretary of State (SofS) to introduce Regulations to set aside the Common Law Duty in certain circumstances. In debate, Lord Hunt (Parliamentary under SofS for Health) described those Regulations as essentially in two parts, with the first part having two specific areas as its focus:

The first part outlines two specific areas of work that require access to patient-identifiable information where, through consultation and consideration by the independent Patient Information Advisory Group, *it has been demonstrated that common law requirements for consent cannot currently be met*. The two areas are: the work carried out by cancer registries to monitor the incidence of cancer and measure mortality and survival rates; and *work to tackle risks to public health such as the communicable disease surveillance and prevention* undertaken by the Public Health Laboratory Service and other contributing agencies.^31^

### B. Health Service (Control of Patient Information) Regulations 2002

The Regulations that made specific provision for disclosure for these areas of work were the Health Service (Control of Patient Information) Regulations 2002. These Regulations allow for Confidential Patient Information^32^ to be disclosed, without patient consent, for defined medical purposes, including the specific support offered (under Regulation 2) to cancer registration and (under Regulation 3) to public health purposes. In addition, in the second part mentioned earlier but not fully described, the 2002 Regulations permit (under Regulation 5) disclosure for medical purposes more broadly defined. The scope of each of these Regulations must be interpreted not only taking the objectives of the instrument as a whole into account, but they must also be read together. The interpretation of each must be undertaken with a thought to the extent to which the other may represent an alternative (and preferable) way of achieving appropriate support for the relevant medical purpose.

In what follows, the content of Regulation 3 in particular will be analysed with a view to clearly setting out (i) the nature of the public health purposes that may be appropriately brought within its scope and (ii) to whom confidential data might be disclosed for these purposes. This is not to say that this will describe the limits of appropriate disclosure, in terms of either scope or recipient, for public health purposes. There remains the possibility that disclosure for public health purposes, for purposes and to persons not within the scope of Regulation 3, may be brought within the scope of the more general Regulation 5, or some other statutory basis for disclosure, or alternatively, justified as appropriate given an overriding ‘public interest’ in disclosure. Despite the importance of Regulation 2 and cancer registration, this will not be further considered here, although it is important to note that not all of the work of PHE needs to be brought within either Regulation 3 or 5.

The advantage of reliance upon a statutory provision, such as either Regulation 2, 3, or 5, is that there is the opportunity for *prospective* clarity on the appropriateness of disclosure of particular kinds. This opportunity, and its associated benefits, can, however, only be realised fully if there is uniform understanding of the scope of the support offered by statute. Here, this includes understanding the limits of both purpose and persons that may rely on Regulation 3 and the implications for potential reliance upon Regulation 5. Clarity regarding the breadth of Regulation 3 is important to describe the controls and safeguards that attach not only to disclosure for particular kinds of purpose but also to particular classes of person. The corollary is greater clarity regarding the controls and safeguards attached to disclosure for particular purposes, to particular persons, under Regulation 5.

### C. Regulation 3: Communicable Disease and Other Risks to Public Health

Subject to a number of qualifications to be considered presently, Regulation 3 of the 2002 Regulations provides a lawful basis for the disclosure of confidential patient information notwithstanding any Common Law Duty of Confidence that might be owed. The Regulations establish a secure legal basis for a health professional to disclose confidential patient information for the purposes of

- diagnosing communicable diseases and other risks to public health;
- recognising trends in such diseases and risks;
- controlling and preventing the spread of such diseases and risks;
- monitoring and managing

- outbreaks of communicable disease;
- incidents of exposure to communicable disease;
- the delivery, efficacy, and safety of immunisation programmes;
- adverse reactions to vaccines and medicines;
- risks of infection acquired from food or the environment (including water supplies);
- the giving of information to persons about the diagnosis of communicable disease and risks of acquiring such disease.

The confidential information must be disclosed to, and processed by, one of a limited number of persons or bodies listed in paragraph 3 of Regulation 3:

- by a public body that has responsibility, as an executive agency of the Department of Health, for public health;
- by persons employed or engaged for the purposes of the health service; or
- by other persons employed or engaged by a Government Department or other public authority in communicable disease surveillance.

Unlike other parts of the 2002 Regulations, Regulation 3 does not require a further decision [by the SofS or the Health Research Authority (HRA)] before it can serve as a lawful basis for disclosure. Put formally, if persons within the scope of paragraph 3 are to process confidential patient information for purposes within the scope of paragraph 2, then Regulation 3 provides a secure legal basis for the disclosure of confidential patient information to those persons for those purposes. The precise scope of both paragraphs 2 and 3, and their interaction, is thus a crucial determinant of the extent of the exception to the general requirement for patient consent that is carved out by Regulation 3 for public health purposes. It has implications for the confidentiality of all patients with records held in England and Wales.

The focus of the argument offered in this article is on the interpretation and scope of just one phrase in paragraph 2: ‘other risks to public health’. However, due to the significance of the interaction between paragraphs 2 and 3, it is relevant context that the list of persons or bodies in paragraph 3 covers a potentially very broad class of persons. In fact, given the nature and arrangement of the comprehensive health service in England and Wales today, and the fact that private companies are regularly contracted to provide services to support the delivery of different aspects of care or treatment, the inclusion within sub-paragraph (b) of persons ‘employed or engaged for the purposes of the health service’ might potentially be very inclusive indeed. It gives rise to a distinct possibility that confidential patient data might be disclosed, for public health purposes, to any party ‘engaged for the purposes of the health service’. This might include private commercial companies. Given current concern over disclosure of confidential patient information to companies that might use that data, even partly for commercial purposes, this is an additional reason to be clear on what processing is permissible within the scope of paragraph 2.^33^

Before turning to consider in more detail the text of paragraph 2, and in particular to ask what public health processing might be brought within the phrase ‘other risks to public health’, there is a separate part of Regulation 3 that deserves brief mention: paragraph 4. This paragraph is worthy of mention at this point not only because it is part of Regulation 3, albeit offering a less trodden path towards lawful data processing than other parts of Regulation 3, but also because it engages safeguards against arbitrary processing that will feature in later analysis *and do not otherwise apply to Regulation 3*. Under 3(4), where it is considered necessary, the SofS may give notice to any body or person specified in paragraph 2 to *require* that person or body to process information. There is no record of this paragraph having ever been invoked. However, were it to be relied upon, then—because it would require express decision by the SofS—the use of Regulation 3(4) would be informed by independent advice.

## III. THE ROLE OF THE INDEPENDENT ADVISORY GROUP

There is an independent advisory group with responsibility for advising on the application of the 2002 Regulations. The group has, with changes in the law, been renamed a number of times through its history. Despite changes, it has existed in some form since it was first called upon to give advice when the Regulations were originally drafted, and it has always had a diverse membership with both expert and lay members. When first created, the group was known as the Patient Information Advisory Group (PIAG). It now operates as a committee of the HRA and is known as the Confidentiality Advisory Group (CAG). In the interim, it was a committee of the National Information Governance Board for Health and Social Care (NIGB), and known as the Ethics and Confidentiality Committee (ECC). Unless referring to a specific piece of historic advice, I will use the generic term the Advisory Group.

The terms of reference of the Advisory Group have changed over the years. Originally, there was a statutory responsibility to consult the Advisory Group before new or amended Regulations were laid before Parliament,^34^ but when NIGB was abolished, this responsibility transferred to the Care Quality Commission and that is where this particular responsibility currently rests.^35^ The Advisory Group has, however, always been invited to advise decision-making under the 2002 Regulations. The Care Act 2014 has, for the first time, created a statutory responsibility to establish a committee for the purposes of giving such advice to decision makers. This would include, but not be limited to, any decision to rely upon Regulation 3(4). The Care Act 2014 requires the HRA to establish a committee for the purposes of giving advice in circumstances where a decision has to be made on whether to permit processing under the 2002 Regulations.^36^ If there is an application to use confidential patient information for research purposes, then the advice is given to the HRA. If for non-research purposes, then it is the SofS. This is why the Advisory Group now has a role advising SofS in relation to Regulation 3(4) but not other parts of Regulation 3: Regulation 3 relates to a non-research purpose, and 3(4) is the only part of Regulation 3 that requires a decision by the SofS. As already stated, if confidential patient information is to be used for the purposes, and by the persons, described in Regulation 3, so long as 3(4) is not to be relied upon, then such persons may rely upon Regulation 3 without making application to CAG to advise SofS.

In contrast to the position under Regulation 3, any use of Regulation 5 requires a decision before the Common Law Duty of Confidence can be set aside and confidential patient information disclosed, without patient consent, for medical purposes. Whether the decision is by the HRA or SofS depends upon whether the application is for a research or non-research use. Any person wishing to establish support under Regulation 5 for particular processing will submit an application to the Advisory Group. The Advisory Group will then provide advice to the relevant decision maker. The application to the Advisory Group will set out the specific purposes of processing, the justification for that processing in terms of improved patient care or public interest and, *inter alia*, why the objective of the processing could not be achieved without reliance upon the Regulations, e.g. through the use of only effectively anonymized data or with patient consent.^37^

Regulation 5 can be used to permit processing for any medical purpose in any of the circumstances set out in Schedule 1 to the 2002 Regulations. Medical purpose is broadly defined to include the purposes of any of the following: ‘preventative medicine, medical diagnosis, medical research, the provision of care and treatment and the management of health and social care services’.^38^ A number of equally broadly defined circumstances are set out in Schedule 1 to the Regulations, and they include processing confidential patient information for the purposes of reducing the identifiability of that data; to conduct medical research that requires access to the past or present geographical location of patients; to identify and contact patients to invite them to participate in medical research; to link information from more than one source; and, to audit, monitor, or analyse the provision of patient care and treatment. Some of the key distinctions between Regulations 3 and 5 may thus be set out in summary form:

**Table:** 

	Regulation 3	Regulation 5
Purpose	(1) (a) diagnosing communicable diseases and other risks to public health; (b) recognising trends in such diseases and risks; (c) controlling and preventing the spread of such diseases and risks.…	Medical purposes: ‘preventative medicine, medical diagnosis, medical research, the provision of care and treatment and the management of health and social care services’ (Section 251 NHS Act 2006).
Recipient	(3) (a) by a public body that has responsibility, as an executive agency of the Department of Health, for public health or (b) by persons employed or engaged for the purposes of the health service; or (c) by other persons employed or engaged by a Government Department or other public authority in communicable disease surveillance.	Any body approved to conduct the processing by the decision maker.
Case-by-case approval required?	No. Not unless there is to be reliance upon 3(4).	Yes. Case-by-case decision on approval following advice from the Advisory Group.
Approval by whom	No specific approval required (unless under 3(4) in which case SofS).	Approval by SofS in the case of non-research medical purposes. Approval by HRA in the case of medical research (also requiring favourable Research Ethics Committee (REC) opinion).

It is possible for processing for the purposes of public health (within Regulation 3) to significantly overlap with the scope of Regulation 5. For example, the survival analysis of patients suffering from *Clostridium difficile* might be regarded as processing for the purpose of effective ‘provision of care and treatment’ (under Regulation 5) or for the purposes of ‘recognising trends’ in communicable diseases and other risks to public health (under Regulation 3). It is important that the interface with Regulation 5 is understood so that applications can be avoided where they would be unnecessary.^39^ Proportionate governance does not require processing for purposes, and by parties, already agreed by Parliament to be appropriate to continue to be subject to repeated case-by-case review, advice, and decision. Furthermore, there are different expectations attached to processing under Regulation 5 as opposed to Regulation 3, and so it is important that the correct route towards lawful processing is consistently followed not only for the sake of efficiency, and proportionate governance, but also predictable regulation.^40^ If the Advisory Group is to appropriately advise on the use of Regulation 5, then they—and the secretariat that supports their activity and acts as the primary filter and source of advice to applicants—will be amongst those that need to have a consistent and uniform understanding of the scope of Regulation 3.^41^ The Advisory Group and their secretariat are not alone in needing this clarity.

Ideally, we require all of those who might potentially receive confidential patient data for public health purposes, as well as all of those who might potentially disclose it to them, to have a consistent and uniform understanding of the applicability of the 2002 Regulations. If we want the level of clarity that will enable transparency and consistent public communication and if we want to avoid disproportionate governance and inconsistent application of the regulatory framework, then we require a clear source of consistent and uniform advice. It needs to be authoritatively clear which aspects of the 2002 Regulations can be relied upon to support disclosure to particular persons for particular public health purposes. Any body within the scope of persons lawfully entitled to process under Regulation 3, i.e. any of those listed under 3(3), needs to have an understanding of the scope of the purposes of processing that fall within 3(2). Not least of all so that they can determine whether it would be appropriate to submit an application to process under Regulation 5. Unfortunately, the language of Regulation 3 is far from unambiguous. In particular, there is scope for different interpretations of the phrase ‘other risks to public health’. What is more, as the Advisory Group has no standing currently to advise on any part of Regulation 3 [other than 3(4)], it is necessary to look for guidance elsewhere on the meaning and scope of Regulation 3 and the scope of this phrase. The view *historically* taken by the Advisory Group, when they did have a broader remit, provides some relevant context and helps to explain why a relatively narrow interpretation of ‘other risks to public health’ has been taken for more than 10 years since the Regulations were first drafted.^42^

### A. ‘Other Risks to Public Health’

The Health Protection Agency (HPA) took over the work of the Public Health Laboratory Service (PHLS) in 2003. At that time, the remit of HPA extended beyond that of the previous PHLS.^43^ In particular, the HPA assumed responsibility for chemical and radiological protection issues for which the PHLS had not been responsible. The PIAG was invited to consider whether the extended remit of the HPA continued to fall within the scope of Regulation 3. At the time, PIAG recognised that the Regulations were ‘broad enough to support work in these areas’.^44^ It was evidently the case that the group considered there to be a sufficiently close analogy between the kind of public health risk posed to a population's health by communicable disease and that presented by the environment risks posed by chemical and radiological hazards. That it *needed* to be a *close* analogy in order to be brought within the terms of Regulation 3 is supported by later reflection by the Advisory Group on the position previously taken: ‘PIAG had advised a narrow interpretation of other risks to public health in relation to Regulation 3, *essentially limited* to chemical and radiation risks and environment emergencies’.^45^

A focus upon chemical and radiological risks and other kinds of environmental hazard that pose a risk to population health might be described as a focus on public health *protection*. In 2011, the Health Select Committee of the House of Commons said that public health practice is now seen as falling into three distinct domains:

- *Health protection*, covering interventions to address environmental threats to the health of the population.
- *Health improvement* (also referred to as health promotion), embracing a very wide variety of areas, including tackling health inequalities and addressing lifestyle issues that impact on health and wellbeing.
- *Health-care public health*, which is concerned with the quality, safety, efficacy, effectiveness, value for money, and accessibility of health-care services.^46^

Each is a serious public health concern, and there will be occasions (e.g. health screening) when it can be difficult to clearly distinguish into which of these ‘three distinct domains’ a particular activity falls. Nevertheless, a distinction in principle between *protection* and *improvement* is recognised, and the activity recognised by PIAG to fall within Regulation 3 can be most closely aligned with the Select Committee's description of health *protection*.^47^

That the Advisory Group did not interpret Regulation 3 to include health *improvement* activity is supported by the position that was taken in relation to the activity of the Public Health Observatories (PHOs). At the same time as the HPA was operating in England and Wales, there were a series of PHOs also operating. The aims and purposes of PHOs were broader than that of the HPA. PHOs ‘produce information, data and intelligence of people's health and health care for practitioners, commissioners, policy makers and the wider community’.^48^ The roles of PHO are described by the Association of PHOs as including ‘monitoring trends in health status and disease; showing how health inequalities are being tackled; assessing the effects of health care interventions, giving Commissioners and service providers the evidence and data they need to reduce inequalities both in access and health outcomes’.^49^

When the NIGB ECC was asked to consider the appropriateness of PHOs relying upon Regulation 3:

Discussion with the applicant had identified that Regulation 3 of the Health Service (Control of Patient Information) Regulations 2002, as originally drafted, had not necessarily taken into account the evolving nature of public health since their establishment. Members acknowledged that it would stretch the boundaries of the current Regulations to bring all of the activities contained within the application within the remit of Regulation 3. The Regulation as originally drafted concerned communicable disease and chemical/biological hazards, and members of the Committee were of the view that PHO activities were not consistent with the current drafting.^50^

Although it was recognised that the nature of the boundary between different kinds of public health activity was evolving, the Advisory Group thought that it would be to go too far to include all of the activities undertaken by PHOs within the scope of Regulation 3.

The work of PHOs, screening programmes, and the HPA has now been brought under the single umbrella of PHE. Need for reliance upon the 2002 Regulations was reduced when the Health Protection (Notification) Regulations 2010 came into force (which cover laboratory flows), but the 2002 Regulations remain an important legal basis for disclosure of confidential patient information to PHE, and others, without patient consent. Now set in statute, the responsibilities of the Advisory Group in relation to Regulation 3 are, however, also limited to advising on Regulation 3(4), i.e. that never-used part of the Regulation mentioned earlier, which allows the SofS to *require* disclosure of confidential patient information. While no body currently has statutory responsibility to advise on the appropriateness of reliance upon other elements of Regulation 3, the processing of confidential patient information for public health purposes was considered as part of the broader Information Governance Review (IGR) chaired by Dame Fiona Caldicott, which produced what has come to be known as the Caldicott2 report.

In March 2013, Caldicott2 described how the 2002 Regulations^51^ provide statutory support for health-care professionals responsible for health protection to know confidential patient information:

For example, during an outbreak of an infectious disease, public health staff may need to identify people who are at risk, perhaps because they have not been vaccinated, or because they have been exposed to an infectious disease or environmental hazard.^52^

While Caldicott2 expressed the view that ‘there was a lack of coherence in regulations across the public health arena’, it noted that ‘Health *Protection* functions are also largely covered by [the 2002 Regulations] but not wider public health functions, as there is no immediate risk to other's health’.^53^ Caldicott2 did, however, further note that ‘[t]hose aspects of public health that relate to health of populations should be reviewed for potential inclusion in Regulation 3 of the Health Service (Control of Patient Information) Regulations 2002’.^54^ Moreover, it was a specific recommendation of Caldicott2 that:

The Secretary of State for Health should commission a task and finish group … to determine whether the information governance issues in registries and public health functions outside health protection and cancer should be covered by specific health service regulations.^55^

The need for clarity on this issue has, therefore, been called for authoritatively. With the Health and Social Care Act 2012 reconfiguring the NHS, and local authorities gaining additional public health responsibility,^56^ there is an urgent need for the review recommended by Caldicott2 and improved clarity on the scope of the existing Regulations. Indeed, the need for further guidance has also been recognised by PHE. Since publication of the Caldicott2 report, PHE has undertaken to lead work with the Department of Health to reduce uncertainty in this area:

Public Health England is working with the Department of Health and the Faculty of Public Health to determine a reasonable interpretation of the phrase ‘other risks to public health’. Further advice will follow once this is resolved.^57^

The remainder of this article is concerned with considering the legal rules, norms, or principles that might constrain or inform that interpretive exercise and to argue that any ambiguity around the scope of ‘other risks to public health’ should be resolved in a particular way. The conclusion to be offered is that the narrow interpretation favoured by the Advisory Group should continue to be preferred so that disclosure for the purposes of public health improvement might be more appropriately managed through Regulation 5. The remainder of the article is intended to justify this conclusion.

## IV. INTERPRETING ‘OTHER RISKS TO PUBLIC HEALTH’

There are a number of basic principles that inform and guide the interpretation of statutes. Any ambiguity in the choice of words used to express parliamentary intention can usually be resolved by reference to the broader legislative context and more general conventions regarding statutory construction. The courts have been increasingly willing to adopt a ‘purposive’ approach to interpretation rather than rely upon a literal interpretation of the words used,^58^ but the fact of certain shared conventions regarding interpretation helps drafters of legislation to anticipate how courts will understand the legislation as drafted beyond an expression of purpose alone:

In relation to interpretation of statutory provisions, [judges] are constrained by rules of language and more or less well-identified background presumptions which condition the way in which it is presumed that Parliament intended a statute to be read.^59^

The basic idea that words should be understood within the context finds specific expression in the principle of interpretation known by the Latin expression ‘noscitur a sociis’: words are known by the company that they keep.^60^ An example of the application of this principle may be seen in the case of *Inland Revenue* v *Frere.*^61^ Here, the court had to consider the meaning of ‘interest’ in the phrase ‘amount of interest, annuities or other annual payments’ in the Income Tax Act 1952. The Court held that short-term interest paid on loans was not tax deductible because other words in the statute made clear that only annual interest was intended to be deductible, and so the word ‘interest’ should not be read in a comprehensive sense despite the lack of any qualifying adjective in the section.^62^ In a similar way here, it is suggested that the phrase ‘other risks to public health’ must be understood within context. One should not extract the phrase ‘other risks to public health’ and reflect in the abstract upon how far the most comprehensive range of risks to public health might extend. There may be no ‘qualifying adjective’ attached specifically to the scope of risks to public health in the second half of the sub-paragraph, but the fact that the relevant phrase is ‘communicable diseases and other risks to public health’ should be understood to limit relevant risks to public health to those similar in kind to the risks posed by communicable disease. The risks that communicable disease poses to an individual are environmental risks to a population; they are immediate and serious risks posed by the external environment to the general public. Protecting people from such risks is a matter of public health protection and not improvement.

The intent and purpose of a statute may also be drawn from an understanding of the state of affairs that existed and was known by Parliament to have existed at the time that it was passed.^63^ Exceptionally, where ambiguity persists, the courts will take into account remarks made in Parliament by promoters of a Bill. The background to the debate surrounding the 2002 Regulations was that the legal basis for the disclosure of confidential patient information for purposes beyond direct care had come under scrutiny. A number of activities were found to be without a clear legal basis; and, a principal aim was to establish a clear legal basis for activity that was already taking place.^64^ Within Parliament, in support of the Regulations, it was recognised that if ‘our *current* surveillance systems are damaged, then there is a real danger of unnecessary cases of infectious disease and even deaths’.^65^ Examples of extant surveillance systems given included ‘the Public Health Laboratory Service's work to monitor infections such as *E. coli* and new variant CJD’.^66^ The motivating state of affairs was thus a risk to existing surveillance systems, which would threaten the ability to provide adequate health *protection* (not improvement).

For parliamentary debate to be taken directly into consideration by a court, a number of conditions originally set out by Lord Browne-Wilkinson in *Pepper* v *Hart* must be satisfied. The conditions are, *inter alia*, (a) the legislation is ambiguous in some respect, (b) statements by a minister or proposer shed light on the ambiguity, and (c) the statements are clear.^67^ Each of these conditions may be satisfied here. Statements in the House of Lords by the Health Minister of the day shed light specifically on the flexibility that the ambiguous phrase ‘other risks to public health’ was intended to allow:

The noble Earl, Lord Howe, raised the issue of why the phrase ‘other public health risk’ is used in the Regulation. He implied that we needed a tighter definition. It is, as noble Lords have suggested, impossible to know in advance what may constitute a risk to public health. The risks that we may face in the future are often unknowable today. Those working to monitor and safeguard public health must be free to act quickly and effectively when a new risk is detected. The alternative is to provide a long list of possible risks that would inevitably fall behind what is needed.^68^

It is clear that any flexibility that can be found within the phrase ‘other risks to public health’ was intended to allow those working to safeguard public health to be ‘free to act quickly and effectively when a new risk is detected’. This is evident in the case of public health risks posed by communicable diseases, chemical spills, or radiation leaks. There is no similar requirement to act quickly when the risks to public health are chronic and posed by factors such as obesity, smoking, or the consumption of alcohol.

While risks to public health posed by chronic environment and lifestyle conditions might be as significant to a population's health in the long term as the immediate risks of communicable diseases, chemical, radiological, and biological hazards, etc., the immediacy of the threat and the consequent need to react quickly—without seeking individual patient consent—are qualitatively different. While there are good reasons to work to improve public health generally, the legal provisions intended to support public health protection may be designed with different safeguards to those required to support public health improvement. The *necessity* and *proportionality* of the interference with privacy when confidential patient information is shared for public health purposes may vary between health protection and health improvement. This is particularly significant when taking into account some of the other legal considerations that will inform and constrain an interpretation of the 2002 Regulations.

### A. Human Rights Act 1998

Since the Human Rights Act 1998 came into force, courts in the United Kingdom have been under a statutory obligation to interpret and give effect to all primary and subordinate legislation, so far as it is possible to do so, in a way that is compatible with a number of the rights recognised by the European Convention on Human Rights (ECHR).^69^ One of the Convention rights incorporated into English Law by the Human Rights Act 1998 is the Article 8 right to a private and family life.

Article 8 recognises that ‘everyone has the right to respect for his private and family life, his home and correspondence’. This right is qualified by a recognition that interference with an individual's enjoyment of the right to a private life may be permitted where it ‘is in accordance with the law and is necessary in a democratic society’. It is well established that the disclosure of confidential patient information, without patient consent, constitutes a prima facie infringement of the right to a private and family life.^70^ The question here is whether disclosure for *broad* public health purposes could be defended as (i) ‘in accordance with the law’, (ii) in pursuit of a legitimate aim, and (iii) ‘necessary’ for the purposes of ‘public safety’, ‘the protection of health or morals’ or for the protection of the rights and freedoms of others.

#### 1. ‘In accordance with the law’

The European Court of Human Rights has interpreted this phrase to not only require the interference in question to have some basis in domestic law but also to be compatible with the rule of law:

The law must thus be adequately accessible and foreseeable, that is, formulated with sufficient precision to enable the individual—if need be with appropriate advice—to regulate his or her conduct. For domestic law to meet these requirements, it must afford adequate legal protection against arbitrariness and accordingly indicate with sufficient clarity the scope of discretion conferred on the competent authorities and the manner of its exercise.^71^

If the phrase ‘other risks to public health’ is given a broad reading, assuming other legal requirements including those imposed by data protection law (discussed in part in the following) are met, then disclosure could have a basis in domestic law under the 2002 Regulations. However, the broader the permissible reading of ‘other risks’, then the more discretion conferred on those parties listed in 3(3). The European Court of Human Rights has found that a ‘law that confers a discretion is not in itself inconsistent with this requirement, provided that the scope for the discretion and the manner of its exercise are indicated with sufficient clarity, having regard to the legitimate aim in question, to give the individual adequate protection against arbitrary interference’.^72^

Thus, if protection against arbitrary interference is an aim, it is a relevant consideration that the narrower the permitted reading of ‘*other risks* to public health’, the smaller the scope for arbitrary interference *and* the more substantial the available safeguards to protect against arbitrary interference.^73^ This is because to narrowly interpret ‘other risks’ is not to deny that activity intended to improve (rather than protect) public health can have a legal basis under the 2002 Regulations. It is only to deny that the basis is Regulation 3. It would remain open for those that wanted to process confidential patient information for the purposes of health improvement to make an application to the Advisory Group to do so under Regulation 5. If an application is submitted for disclosure under Regulation 5, then reasons for the disclosure must be provided as part of the application, those reasons are subject to independent review, and the advice on their adequacy is published in the Advisory Group minutes.

Consequently, permitting disclosure for public health improvement purposes under Regulation 5, rather than Regulation 3, better meets the requirements of the ‘rule of law’. The additional protections associated with decisions to permit disclosure under Regulation 5 provide protections against arbitrary interference.

#### 2. ‘In pursuit of a legitimate aim’

There is nothing within Article 8(2) of the Convention to limit the nature of the ‘protection of health’. If ‘other risks’ even given the broadest reading *do* present a risk to public health as a matter of fact, then one might argue that disclosure for the purposes of addressing those risks falls squarely within a specified legitimate aim. The nature of the risk, and the need to address it through the particular measures proposed, is assessed through the tests of necessity and proportionality.

#### 3. ‘Necessary in a democratic society’

As well as in pursuit of a legitimate aim, in order to be consistent with Article 8 ECHR and the requirement that disclosure is *necessary* in a democratic society, the reasons for interference must be relevant and sufficient and the interference itself *proportionate* to the need:^74^

An interference will be considered ‘necessary in a democratic society’ for a legitimate aim if it answers a ‘pressing social need’ and, in particular, if it is proportionate to the legitimate aim pursued and if the reasons adduced by the national authorities to justify it are ‘relevant and sufficient’.^75^

The European Court on Human Rights (ECtHR) grants member states a certain margin of appreciation when determining whether an action is ‘necessary’ for the purposes of achieving a legitimate aim. Courts in member states are often considered best placed to determine whether an interference is necessary,^76^ and ‘it is therefore primarily the responsibility of the national authorities to make the initial assessment as to where the fair balance lies in assessing the need for interference in the public interest with individuals’ rights under art.8 of the Convention’.^77^

But, states do not have ‘an unlimited power of appreciation’ and ‘the domestic margin of appreciation thus goes hand in hand with a European supervision’.^78^ How closely the ECtHR will review such decisions will depend upon the subject matter,^79^ but the proportionality of the interference may be doubted if a less intrusive alternative could achieve the same objective. In *Hatton* v *United Kingdom* (2003), the ECtHR:

underlined that in striking the required balance States must have regard to the whole range of material considerations. … States are required to minimise, as far as possible, the interference with Art.8 rights, by trying to find alternative solutions and by generally seeking to achieve their aims in the least onerous way as regards human rights.^80^

To reiterate an important point, a narrow reading of Regulation 3 would not prevent disclosure for the purposes of addressing ‘other risks’ to public health, read broadly; it would only require consideration of such disclosures under Regulation 5 instead. The ECtHR has established that one of the things that will be especially material when determining whether a respondent state has remained within their margin of appreciation is ‘the procedural safeguards available to the individual’.^81^ Notably, there are more robust procedural safeguards associated with Regulation 5 than with Regulation 3.^82^ Reliance upon Regulation 5 is subject to case-by-case consideration by either the HRA or the SofS. Any decision on whether to support disclosure would be made with the benefit of independent advice. The minutes of the Advisory Group are published on the HRA's website, and they include records of the deliberations made in relation to all applications and the reasons for the Advisory Group's recommendation to the HRA and the SofS. Transparency in relation to processing under Regulation 5 is further improved due to the fact that under Regulation 6, any transfer of confidential patient information between persons under Regulation 5 must be recorded in a public register. There is no such requirement in relation to processing under Regulation 3.

While, therefore, there is no guarantee that a challenge to the Convention compatibility of using Regulation 3 would necessarily succeed, there are nonetheless far stronger, defensible reasons to rely on Regulation 5.

## V. PATIENT DISSENT AND ADDITIONAL CONDITIONS

If processing were to take place under Regulation 5 rather than Regulation 3, it would also be subject to any additional conditions that the decision maker attached to that processing on the recommendation of the Advisory Group. This typically includes the condition that although consent is not required, any patient dissent to processing must be respected.^83^ The use of confidential patient information for indirect care purposes, in the face of express patient objection, will be supported only in the most exceptional circumstances, e.g. ‘serious public safety concerns’.^84^ In order to protect patient trust in the confidentiality of the health service, the Advisory Group has thus advised respecting dissent in circumstances that go further than the Data Protection Act 1998 requires.^85^ The effect of their advice has been to give a patient more control over the use of his or her information than would otherwise be available under the law.

There is a right to prevent processing contained within Section 10 of the Data Protection Act 1998, but the circumstances in which a data controller must respect any objection to the processing of personal data by a data subject are limited. Section 10(1) of the Data Protection Act 1998 states that an individual is entitled to require a data controller to cease, or not to begin, processing any personal data of which he or she is a data subject on the grounds that:

- the processing of those data or their processing for that purpose or in that manner is causing or is likely to cause substantial damage or substantial distress to him or to another, and
- that damage or distress is or would be unwarranted.

The condition typically recommended by the Advisory Group requires patient dissent to be respected in *all* cases; not only those where processing is causing or is likely to cause *unwarranted substantial* damage or *substantial* distress. Objections motivated by other concerns, i.e. other than concerns for substantial damage or substantial distress, could not be effectively expressed through Section 10. However, they typically would be respected if processing were supported under Regulation 5 of the 2002 Regulations.

It is arguably more consistent with recent commitments regarding patient choice^86^ to restrict a patient's right to object to processing to the more limited range of circumstances set out in Section 10 only when the processing is for the purposes of health protection rather than improvement. When processing is for the purposes of health improvement, then it was the view of Caldicott2 that when data were not being used in an anonymized form, it should be treated like research from an information governance perspective.^87^ If data were being processed under the 2002 Regulations for research purposes, then it would be processed under Regulation 5 (and not Regulation 3) and the additional safeguards, including the requirement that patient dissent is typically respected, would apply. It may also provide additional reassurance that data were only to be used in ways that individuals would consider appropriate.

The value of the deliberative potential inherent to the operation of the advisory group should not be underestimated. Recruitment to the group is via open competition and comprises both expert and lay members with a broad range of experience. The deliberations of a group with broad-based membership create a pressure to ‘facilitate decisions that have the quality of justifiability’.^88^ Members are aware that their deliberations are recorded in minutes that are publicly accessible. This may promote the democratic legitimacy of the decision in a way that the structure of Regulation 3 does not allow.^89^ What is more, when considering whether a proposal is ‘in the public interest’, the Advisory Group considers that:

as a basic principle, the more readily that one can anticipate acceptance for a proposal across a broad range of ‘user groups’—e.g. if one can anticipate acceptance not only from those that might benefit from the processing but also those whose data is to be processed without consent—then the clearer the indication, that the processing is, at least prima facie, in the public interest. The CAG is thus seeking to anticipate the ‘reasonable acceptability’ of a proposal by patients and public.^90^

The suggestion is that independent impartial advice, motivated to apply this understanding of ‘public interest’, may contribute towards decisions being taken, and being seen to be taken, for reasons ‘justifiable to those affected’.^91^ It is consistent with the ambition that we move towards greater consistency, proportionality, and transparency and that, ultimately, confidential patient data are only used for purposes that patients have reason to both accept and expect.

## VI. CONCLUSION

It is important that we allow ourselves, as a society, to reap the benefits of advances in information technology and allow ourselves the public health advantages that might follow the disclosure of confidential patient information for public health analysis. However, if that disclosure is to be without an individual's explicit consent, then we need to be confident that data only flow on a secure legal basis, with appropriate and proportionate controls and safeguards, transparently in circumstances that patients can recognise to be appropriate.

Alternative interpretations of the law result in different safeguards being attached to disclosure. It is important now to establish the legal bases for disclosure of confidential patient information for different purposes. Without that clarity, we cannot achieve the relevant transparency and engage in the urgently needed public dialogue regarding the adequacy of current controls and safeguards and the opportunities associated with the use of confidential patient data for purposes beyond direct patient care. The Health Service (Control of Patient Information) Regulations 2002 establish a legal basis for data to be disclosed for public health purposes without patient consent. Under the Regulations, there is, however, more than one potential route towards lawful processing. The alternatives have different safeguards and conditions attached. There are relative merits and demerits attached to each. If one processes under Regulation 3, then there is no need to go through the administrative inconvenience and delay of applying for support for that processing. When processing for the purposes of health protection, the immediacy of the support available has obvious advantages. Delay could cost lives.

It is equally true that a failure to process information for purposes of health improvement might also lead to lives being lost. There are chronic and significant health effects attached to various lifestyle and environment factors. Some of these are beyond individual control. Many of these are experienced disproportionately by certain sections of society.^92^ A reduction in inequality, as much as an improvement in health, requires access to confidential health information for public health purposes. However, when access is for health improvement purposes, while access itself may be no less justified, access under Regulation 3 is not the most appropriate or suitable legal basis for disclosure without patient consent. There is no justification for discarding the additional safeguards associated with processing under Regulation 5. It should be clear that if confidential patient information is being disclosed for the purposes of public health improvement, then the most appropriate basis under the 2002 Regulation is Regulation 5. This requires an application to be made to the Independent Advisory Group and allows for the necessity and proportionality of the interference with individual privacy to be subject to scrutiny by a panel of persons, expert, and lay, drawn from a wide variety of backgrounds. The reasons for any recommendation of support are published within the minutes of Advisory Group meetings, and any disclosure, authorised by the SofS for Health, is recorded in a public register. None of these things need to happen if disclosure is under Regulation 3.

In addition, a narrow interpretation of the phrase ‘other risks to public health’ contained in Regulation 3, restricted to the purposes of health protection, is more consistent with the principle that words should be understood within the context, that statutes are purposively interpreted consistent with the intentions of parliament, and that they are read in a way that is consistent with obligations recognised by and imposed under the Human Rights Act 1998. Access to confidential patient information for public health purposes will be more transparent, robust, and deserving of public confidence if, when individual consent is not practicable, that impracticability is tested under Regulation 5. As the future of data protection law is now debated, it is particularly important to demonstrate that it *is* possible to provide access to identifiable information without individual consent and still appropriately protect an individual's right to respect for their private life.

## DECLARATIONS

Mark Taylor is currently Chair of the CAG for the HRA. This committee provides independent advice on the application of the Health Service (Control of Patient Information) Regulations 2002. He is also member of a number of other groups and committees with an interest in the protection of patient privacy and issues regarding health research access to patient data. The views expressed in this article are entirely personal and expressed in the capacity of an academic at the University of Sheffield. They should not be attributed to any other organisation or body.

## Footnotes

1 We have seen the response to proposals to require GPs to disclose patient records to the Health and Social Care Information Centre so that they might be linked with other health-care records for various secondary purposes. For discussion of the care.data programme see M Taylor, ‘Information Governance as a Force for Good? Lessons to be Learnt from Care.data’, (2014) 11(1) SCRIPTed 1 <http://script-ed.org/?p=1377>.

2 Ipsos Mori, *Public Attitudes to the Use and Sharing of Their Data.* (July 2014, Research for the Royal Statistical Society by Ipsos Mori). <http://www.statslife.org.uk/files/perceptions_of_data_privacy_charts_slides.pdf>; Davidson, McLean, Treanor, Aitken, Cunningham-Burley, Laurie, Pagliari, and Sethi ‘Public Acceptability of Data Sharing between the Public, Private and Third Sectors for Research Purposes’ *Scottish Government Social Research* (2013) <http://www.scotland.gov.uk/Resource/0043/00435458.pdf>.

3 There is reason to believe that the public are willing to accept access to confidential health information for secondary purposes without explicit consent as appropriate, if certain conditions are met, even if this would not be their preference. See M.J. Taylor and N. Taylor ‘Health Research Access to Personal Confidential Information in England and Wales: Assessing Any Gap in Public Attitude between Preferable and Acceptable Models of Consent’, (2014) 10(15) *Life Sciences, Society and Policy* <http://www.lsspjournal.com/content/10/1/15>.

4 Information Governance Review, *Information: To Share or Not to Share* (March 2013), 87.

5 A concern that has been recognised by the courts. See, e.g. *X Health Authority* v *Y* [1988] 2 ALL ER 648; *H (a Healthworker)* v *Associated Newspapers Ltd.* [2002] EWCA civ 195; *R* (*on the application of Sue Axon*) v *Secretary of State for Health* [2006] EWHC 37 (Admin), [7].

6 Department of Health *Confidentiality: NHS Code of Practice* (November, 2003), 11.

7 The need for a more transparent dialogue regarding existing and potential uses of patient data for secondary purposes has also been recognised in the USA. See, C. Safran, M. Bloomrosen, W. E. Hammond, S. Labkoff, S. Markel-Fox, P. C. Tang, and D. E. Detmer, **‘**Toward a National Framework for the Secondary Use of Health Data: An American Medical Informatics Association White Paper’ (2007) January–February; 14(1) *J Am Med Inform Assoc* 1–9 <http://www.ncbi.nlm.nih.gov/pmc/articles/PMC2329823/>; also, the Canadian academies are currently conducting a review of best practices in health- and social-care data sharing: <http://www.scienceadvice.ca/en/assessments/in-progress/health-data.aspx>.

8 Concerns about the consequences of the Health and Social Care Act 2012, and the extended powers of the Health and Social Care Information Centre, led *inter alia* to the establishment of the pressure group ‘MedConfidential’ <http://medconfidential.org/>. MedConfidential are campaigning for the ‘consensual, safe and transparent’ flow of data into, across, and out of the NHS.

9 M. Taylor and B. Thompson ‘Update on the European Data Protection Regulation’ (Briefing Paper, (2013) [38], available from <http://p3g.org/programmes/elsi-20>.

10 Any disclosure of confidential patient information must be consistent with the requirements of the Data Protection Act 1998. As long as data are processed in ways consistent with the data protection principles set out in Part I, Schedule 1, of the 1998, there is nothing in the Data Protection Act 1998 from preventing the disclosure or other processing of confidential patient data for public health purposes without patient consent. Confidential patient information, so far as it relates to the living, will be ‘sensitive personal data’. It will, therefore, be necessary (in order to satisfy the first data protection principles of ‘fair and lawful’ processing) to satisfy at least one of the conditions set out in Schedule 2 and at least one of the conditions set out in Schedule 3 of the 1998 Act. A Schedule 2 condition can be satisfied by demonstrating that the processing is necessary for the ‘exercise of any function of a public nature exercised in the public interest by any person’ [5(d)] or is necessary ‘for the purposes of legitimate interests pursued by the data controller … except where the processing is unwarranted in any particular case by reason of prejudice to the rights and freedoms or legitimate interests of the data subject’ [6(1)]. A Schedule 3 condition can be satisfied by demonstrating that the processing is necessary for medical purposes and is undertaken by a health care professional or person owing an equivalent duty of confidence (8).

11 See open letter to Chief Executive, Director of Public Health from Prof. John Newton, CKO, Public Health England, 24 May 2013.

12 HRA CAG Principles of Advice (April 2013), 1.4.1, 6 <http://www.hra.nhs.uk/documents/2013/09/v-2_principles_of_advice_-_april_2013.pdf> accessed 9 December 2014.

13 Independent review and individual opt-out were two of the safeguards described by patients in relation to research use of their data as reasons for accepting a model of consent, e.g. opt-out, other than that which might be their preference. See M.J. Taylor and N. Taylor ‘Health Research Access to Personal Confidential Information in England and Wales: Assessing Any Gap in Public Attitude between Preferable and Acceptable Models of Consent’ (2014) 10(15) *Life Sciences, Society and Policy* <http://www.lsspjournal.com/content/10/1/15>. While that study was focussed upon research rather than public health, it may be expected that these safeguards might also have the advantage of supporting public confidence in appropriate processing.

14 M. Dixon-Woods, P. Carter, and G. Laurie ‘The Social Licence for Research: Why Care.data Ran into Trouble’ *J Med Ethics* doi:10.1136/medethics-2014-102374 <http://jme.bmj.com/content/early/2015/01/23/medethics-2014-102374.short?g=w_jme_top10_tab>.

15 A GP would traditionally have been the ‘family doctor’. Although the service and role of the GP have changed over the years, it remains the case that most NHS patients will be registered with a GP who will typically act as a first point of contact, advice, and specialist referral in all but emergency situations.

16 See NHS England *Confidentiality Policy* (NHS England, June 2014) <http://www.england.nhs.uk/wp-content/uploads/2013/06/conf-policy-1.pdf>; Department of Health *Confidentiality: NHS Code of Practice* (7 November 2003) <https://www.gov.uk/government/publications/confidentiality-nhs-code-of-practice>.

17 It has been established that the duty only extends to identifiable data, see *R v Dept of Health, ex p Source Informatics* [2000] 1 All ER 786.

18 This has been expressed most recently in the context of sharing support care [see Information Governance Review *Information: to share or not to share* (March 2013)], but there has been a long-standing concern within the research community that information governance can be a disproportionate impediment to appropriate health research. See, for example, Academy of Medical Sciences, *A New Pathway for the Regulation and Governance of Health Research* (January 2011) and also Academy of Medical Sciences, *Personal Data for Public Good: Using Health Information in Medical Research* (January 2006) <http://www.acmedsci.ac.uk/viewFile/publicationDownloads/newpathw.pdf>.

19 *Campbell* v *MGN Limited* [2004] UKHL 22, [22] *per* Lord Nicholls; *W* v *Edgell* [1989] EWCA Civ 13.

20 Department of Health, *Confidentiality: NHS Code of Practice* (November 2003), p7 <https://www.gov.uk/government/publications/confidentiality-nhs-code-of-practice>. Although one should note that in *R* (*on the application of W, X, Y, and Z*) v *The Secretary of State for Health* v *The Secretary of State for the Home Department* [2014] EWHC 1532 (Admin) Mr Justice Silber in the High Court found that the disclosure of identifying information, if omitting details of an individual's illness or medical history, may not be confidential. Detailed analysis of this decision is not necessary for the purposes of the argument offered here because disclosure for public health purposes will invariably require disclosure of some clinically significant information alongside the detail enabling an individual's identification.

21 A rather pragmatic distinction is currently drawn in practice between a local clinical audit that is conducted by those that provide a patient's care and those that support them, and an audit, e.g. a national audit, that requires the disclosure of confidential patient information outside of an organisation. See GMC *Confidentiality: Guidance for Doctors* (October 2009), 13–14. It should be noted that in either case, the NHS Code of Practice on Confidentiality states that it ‘extremely important that patients are made aware of information disclosures that must take place in order to provide them with high quality care. In particular, clinical governance and clinical audits, which are wholly proper components of health care provision, might not be obvious to patients and should be drawn to their attention’. Ibid, p7.

22 Doctors are encouraged to inform patients with whom they will be sharing confidential information, even for care purposes, in order to avoid surprises and to ensure that any patient consent is appropriately informed. GMC *Confidentiality: Guidance for Doctors* (October 2009), 12; The ‘culture of caution’ around data sharing and linkage is further discussed by N. Sethi and G. Laurie ‘Delivering Proportionate Governance in the Era of eHealth’ *Medical Law International* (June 2013) 13(2–3), 168–204.

23 *X Health Authority* v *Y* [1988] RPC 379.

24 *Attorney-General* v *Guardian Newspapers Ltd.* (No. 2) [1990] 1 A.C. 109, per Lord Goff of Chieveley at p282.

25 [1989] EWCA Civ 13.

26 See *W* v *Edgell* [1989] EWCA Civ 13 *per* Lord Bingham at p424 [E–G].

27 In that case, it was the judgment of both Sir Stephen Brown (p416 [E]) and Lord Bingham (p421[E]) (agreed by Sir John May) that the disclosure fell within the circumstances of paragraph 81(g) set out in the then current edition of the GMC's ‘Blue Book’: ‘Rarely, disclosure may be justified on the ground that it is in the public interest which, in certain circumstances such as, for example, investigation by the police of a grave or very serious crime, might override the doctor's duty to maintain his patient's confidence.’ cited by Lord Bingham at p421 [E].

28 *Fraser* v *Evans* [1969] 1 Q.B. 349, 362 (*per* Lord Denning).

29 [1979] 344 Chancery, 183.

30 *Attorney-General* v *Guardian Newspapers Ltd.* (No. 2) [1990] 1 A.C. 109, *per* Lord Goff of Chieveley at 282.

31 HL Deb, 21 May 2002, Vol. 635 cc725–58, 727 (emphasis added).

32 Confidential patient information is a sub category of patient information. The National Health Service Act 2006 defines patient information as ‘(a) information (however recorded) which relates to the physical or mental health or condition of an individual, to the diagnosis of his condition or to his care or treatment, and (b) information (however recorded) which is to any extent derived, directly or indirectly, from such information, whether or not the identity of the individual in question is ascertainable from the information’ [s251(10)]. Patient information is ‘confidential patient information’ where ‘(a) the identity of the individual in question is ascertainable (i) from that information, or (ii) from that information and other information which is in the possession of, or is likely to come into the possession of, the person processing that information, and (b) that information was obtained or generated by a person who, in the circumstances, owed an obligation of confidence to that individual’ [s251(11)].

33 This concern has been expressed, *inter alia*, by the House of Commons Health Select Committee, in relation to disclosures by the NHS Information Centre of patient level (though not identifiable) data to commercial companies. See, for example, House of Commons Health Committee, Oral evidence: Handling NHS patient data, HC 484 (Tuesday 1 July 2014), Q438 <http://data.parliament.uk/writtenevidence/committeeevidence.svc/evidencedocument/health-committee/handling-of-nhs-patient-data/oral/11192.html>.

34 The responsibility to consult the PIAG was originally contained in S61 of the Health and Social Care Act 2001. The responsibility to consult NIGB was under Section 252 of the National Health Service Act 2006 prior to amendment.

35 See S252 NHS Act 2006 as amended.

36 ‘The HRA must appoint a committee for the purpose of giving advice—(a) to the HRA in connection with the exercise of the HRA's function under regulation 5(1)(a) of the Health Service (Control of Patient Information) Regulations 2002 (S.I. 2002/1438) (approval for processing confidential patient information); (b) to the Secretary of State in connection with the exercise of the Secretary of State's functions under regulations 2, 3(4) and 5 of those Regulations (processing of confidential patient information)’, paragraph 8(1), Schedule 7.

37 A pre-application check list setting out the kinds of things that are taken into consideration by the Advisory Group is available on the HRA website. See, in particular questions 6–11 that are intended to ‘reflect the minimum legal criteria set out in legislation’ HRA CAG, ‘CAG Pre Application Decision Tool’ (HRA, undated) <http://www.hra.nhs.uk/documents/2013/08/cag-pre-application-decision-tool.pdf> (last accessed 28 July 2013). There is also a document setting out some of the principles that guide the advice given HRA CAG ‘Principles of Advice: Exploring the concepts of “Public Interest” and “Reasonably Practicable”’ (Improvement Programme Series of Papers, April 2013) <http://www.hra.nhs.uk/documents/2013/09/v-2_principles_of_advice_-_april_2013.pdf>.

38 S251(12) NHS Act 2006.

39 In the event, this kind of activity was considered by the ECC to appropriately fall within the scope of Regulation 3, and not Regulation 5, and so an application was not necessary. See discussion relating to survival analysis of a cohort of *C.**difficile*-infected and non-infected patients admitted to Addenbrooke's hospital between 2005 and 2007. ECC Meeting minutes 24 November 2009, p17–18.

40 Proportionate governance is a recurring theme in the Nuffield Council of Bioethics' ‘The collection, linking and use of data in biomedical research and health care: ethical issues' (February, 2015), see especially 6.44 <http://nuffieldbioethics.org/project/biological-health-data/> See also Sethi and Laurie, Footnote 22.

41 Currently, it is PHE who responds to queries regarding whether particular purposes and parties fall within the scope of Regulation 3.

42 Under S61(3) of the Health and Social Care Act 2001, there was a general provision permitting the SofS to ‘seek the views of the Advisory Group on such other matters connected with the processing of patient information or of any information (other than patient information) obtained or generated in the course of the provision of the health service as he considers appropriate’.

43 History of the Health Protection Agency 2003–2013: Witness Seminar <http://www.hpa.org.uk/webc/HPAwebFile/HPAweb_C/1317138879311>.

44 PIAG meeting minutes, Tuesday 10 June 2003, para. 5.8.

45 CAG minutes, 14 June 2013, 6 (emphasis added).

46 House of Commons Health Committee ‘Public Health’ Twelfth Report of Session 2010–2012 Volume 1: Report, together with formal minutes, 3, <http://www.publicinformationonline.com/secure/d27128.pdf>.

47 Similar distinct domains of public health were also described by the Information Governance Review, *Information: To Share or Not to Share* (March 2013), 85.

48 Association of Public Health Observatories Website <http://www.apho.org.uk/default.aspx?QN=ABOUT_DEFAULT>.

49 Ibid.

50 ECC minutes 28 September 2010, 10.

51 Alongside the Health Protection (Notification) (and related) Regulations 2010.

52 Information Governance Review, 85.

53 Information Governance Review, 87 (emphasis added).

54 Ibid.

55 Recommendation 13; Information Governance Review, 88.

56 Local authorities public health responsibilities (England)—Common Library Standard Note available at <http://www.parliament.uk/business/publications/research/briefing-papers/SN06844/local-authorities-public-health-responsibilities-england>.

57 Open letter to Chief Executive, Director of Public Health from Prof John Newton, CKO, Public Health England, 24 May 2013. <https://www.gov.uk/government/publications/council-access-to-health-care-data-from-the-nhs-letter-from-the-cko>.

58 See *R* v *Secretary of State for Health* ex parte *Quintavalle* (on behalf of Pro Life Alliance) [2003] UKHL 13, especially [6–10] per Lord Bingham.

59 P. Sales, ‘Judges and Legislature: Values into Law’ *Cambridge Law Journal* (2012) 71(2), 287–296, at p291.

60 See seminally *Muir v Keay* (1874–75) L.R. 10 QB 594.

61 [1964] 3 All ER 796.

62 See speech of Viscount Radcliffe, at 427.

63 See *Royal College of Nursing of the United Kingdom* v *Department of Health and Social Security* [1981] AC 800, 822 per Lord Wilberforce (dissenting). Note that Lord Bingham suggested that this passage ‘may now be treated as authoritative’ in *Quintavalle.*

64 ‘For many years, the NHS has been run along paternalistic lines, with managers and clinicians making decisions about what is best for patients with little or no input from patients and their families… We need to bring services in line with the expectations of patients, with the requirements of law and with the high standards that we have set as our goals… But it is important to acknowledge that it will take time to implement those changes. We cannot simply stop medical research or clinical audit, abandon all the valuable work on cancer that is underway or leave aside our responsibilities to monitor and sustain public health while we take time out to build systems and devise ways of working that meet the standards that we now know should apply… So we have to manage change in a way that supports essential activity while the building blocks for improvement are put in place. We cannot risk some of the most important health service work programmes grinding to a halt because they are unable at present to meet required standards...’ HL Deb, 21 May 2002, Vol. 635, cc725–726.

65 Ibid, 727 (emphasis added).

66 Ibid. Note, however, that the monitoring of CJD was in fact undertaken in Scotland and not by PHLS.

67 See the dicta of Lord Browne-Wilkinson in *Pepper* v *Hart* [1993] AC 593; [1993] 1 ALL ER 42, 69.

68 HL Deb, 21 May 2002, Vol. 635, cc752.

69 S.3 Human Rights Act 1998.

70 *MS* v *Sweden* (1997) 45 BMLR 133; *Z* v *Finland* (1997) 45 BMLR 107.

71 *Colon* v *the Netherlands* (2012) 55 EHRR SE5 [72].

72 *Hachette Filipacchi Associates* v *France* (2009) 49 EHRR 23, 522–523 [31].

73 There would be the possibility of an action for judicial review if a public body was suspected of applying its powers in an arbitrary way. There are also certain safeguards contained within Regulation 7 that apply to Regulation 3. Note that the possibility of judicial review has not been sufficient to persuade the ECtHR that there is adequate protection from arbitrary exercise of power (See *Gillan* v *UK* (2010) 50 EHRR 45, at [80]).

74 *Handyside* v *UK* (1979–1980) 1 EHRR 737, 753–755.

75 *Colon* v *the Netherlands* (2012) 55 EHRR [88].

76 National authorities have direct democratic legitimation, see *Hatton* v *United Kingdom* (2003) 37 EHRR 28 at 97; *Handyside* v *UK* (1979–80) 1 EHRR 737 at [48].

77 *Colon* v *the Netherlands* (2012) 55 EHRR SE5 [87].

78 *Handyside* v *UK* (1979–1980) 1 EHRR 737, 754.

79 See J. Rivers ‘Proportionality and Variable Intensity of Review’ (2006) 65(1), CLJ 174 and *Huang v Secretary of State for the Home Department*.

80 *Hatton* v *UK* (2003) 37 EHRR 28 [86]. See also paragraphs 86–104, 125, 128, and 129.

81 *Connors* v *UK* (2005) 40 EHRR 9 at [83].

82 The application of more robust procedural safeguards may also increase the likelihood that it will be considered to be consistent with other international norms: ‘Data subjects should be guaranteed the right to information about the nature and objectives of the data collection, and the methods to be used, as well as their rights in regard to the data stored and processed subsequent to collection. In particular, and subject to the existence of other effective safeguards laid down by domestic law, the express and informed consent of the data subjects should be sought before data can be collected and used’, paragraph ii, Council of Europe, Committee of Ministers, Recommendation No. R(89) 4 on Collection of Epidemiological Data on Primary Health Care (6 March 1989).

83 HRA CAG ‘Principles of Advice: Exploring the concepts of “Public Interest” and “Reasonably Practicable”’ (Improvement Programme Series of Papers, April 2013), 6 <http://www.hra.nhs.uk/documents/2013/09/v-2_principles_of_advice_-_april_2013.pdf>.

84 Ibid.

85 S251(12) NHS Act 2006.

86 L. Evenstad ‘Hunt pledges to respect patient data’ *eHealth Insider* 26 April 2013 <http://www.ehi.co.uk/news/ehi/8549/hunt-pledges-to-respect-patient-data>.

87 Information Governance Review, 86.

88 C. Mullen, D. Hughes, and P. Vincent-Jones, ‘The Democratic Potential of Public Participation: Healthcare Governance in England’ *Social & Legal Studies* (2011) 20(1), 21–38, 25 <http://sls.sagepub.com/content/20/1/21>.

89 ‘[D]emocratic legitimacy might be claimed where participation leads to reasoned decisions that are justifiable to the public in the relevant constituency’. Ibid, 24–25.

90 HRA CAG, ‘Principles of Advice: Exploring the concepts of “Public Interest” and “Reasonably Practicable”’ (Improvement Programme Series of Papers, April 2013), 3 <http://www.hra.nhs.uk/documents/2014/12/principles-advice-april-2013-v-2.pdf>.

91 Mullen, Hughes, and Vincent-Jones, above n 88, 28.

92 HM Government ‘*Health Lives, Health People: Our Strategy for Public Health in England*’ (November 2010) (Cmd 7985), esp. p15.

